# Physiological mechanism underlying the effect of high temperature during anthesis on spikelet-opening of photo-thermo-sensitive genic male sterile rice lines

**DOI:** 10.1038/s41598-020-59183-0

**Published:** 2020-02-10

**Authors:** Jing Chen, Yangdong Xu, Keqi Fei, Rui Wang, Jiang He, Lidong Fu, Shimei Shao, Ke Li, Kuanyu Zhu, Weiyang Zhang, Zhiqin Wang, Jianchang Yang

**Affiliations:** Jiangsu Key Laboratory of Crop Genetics and Physiology/Co-Innovation Center for Modern Production Technology of Grain Crops, Yangzhou University, Yangzhou, Jiangsu China

**Keywords:** Plant physiology, Plant sciences

## Abstract

Decrease in the grain yield resulted from a low percentage of opened spikelets under high temperature (HT) during anthesis is a serious problem in the seed production of photo-thermo-sensitive genic male sterile (PTGMS) rice (*Oryza sativa* L.) lines, and the mechanism is little understood. Elucidating the physiological mechanism underlying the effect of HT during anthesis on spikelet-opening of PTGMS lines would have great significance in exploring the effective way to mitigate the adverse effect of HT. In this study, two PTGMS lines and one restorer line of rice were used and were subjected to normal temperature (NT) and HT treatments. The results showed that, compared with NT, HT significantly decreased the percentage of opened spikelets, fertilization percentage and seed-setting by significantly increasing the percentage of wrapped spikelets and reducing the spikelet-opening angle, length of spikelet-opening time. The HT significantly decreased the contents of soluble sugars, jasmonic acid (JA) and methyl jasmonate (MeJA) in the lodicules before and at glume-opening, which were significantly correlated with and accounts for the low percentage of opened spikelets under HT for rice, especially for the PTGMS lines.

## Introduction

Male sterility in cereals has played a critical role in the utilization of heterosis by facilitating hybrid breeding and greatly contributed to the increase in productivity for many crops such as rice (*Oryza sativa* L). The establishment of the breeding theory and technology of the “two-line method” hybrid rice (hereinafter referred to as the two-line hybrid rice), especially photo-thermo-sensitive genic male sterile (PTGMS) rice lines, is another major scientific and technological innovation in the utilization of heterosis after the “three-line method” hybrid rice application^1–7^. Compared with three-line hybrid rice with nuclear-plasma interaction pollen sterility as the core technology in a hybrid rice system, two-line hybrid rice can easily maintain sterile line production without using maintainer lines. Moreover, two-line hybrid rice also has many other advantages, such as a wider range of germplasm resources which can be used as breeding parents, greater heterosis, and simpler procedures for breeding and hybrid seed production^2,3,7–10^. Today, the annual planting area of two-line hybrid rice in China has exceeded 5 million hectares, which has become a main way using heterosis in rice^3,11–15^. However, the fertility of PTGMS rice line is easily affected by temperature and light. Usually, the PTGMS rice line tends to produce low purity of hybrid seeds due to self-cross at a low temperature (23–24 °C) in the seed production. The spikelets of PTGMS lines during anthesis could not open normally at high temperature (HT, ≥35 °C), which seriously reduces the hybrid seed yield^2,3,11–13,16^.

Temperature is an important ecological factor for plant growth. HT (≥35 °C) could seriously influence the growth and development of rice, at either vegetative or reproductive growth stage^17–21^. With the increase in global greenhouse effect, HT stress has become a major hazard in rice production including “two-line” hybrid rice seed production^5,20,22,23^. Rice is generally more sensitive to HT at the reproductive stage, especially at anthesis, than at the vegetative stage^18,24–27^. HT at anthesis could seriously decrease grain yield by reducing spikelet-opening and seed-setting percentage^28,29^. HT at anthesis even over 1 h could result in pollen sterility^30^. What’s more, the anther indehiscence, failure in swelling of the pollen grains, abnormal growth of pollen grains, along with hormone imbalance, especially the increase in Ethylene, auxin and other change in hormone such as the decrease in polyamine, which all caused by HT at anthesis, played a non-ignorable role in contributing to spikelet sterility to some extent^25,31,32^. HT (≥35 °C) at grain filling stage could inhibit the activity of key enzyme in the sucrose-starch metabolic pathway in grains and as a result, influencing the grain filling process, inducing low seed-setting, grain weight and poor rice quality^33,34^. However, previous studies have mostly focused on conventional rice varieties because of it’s sensibility in response to high temperature of male organ, grain pollen^30,35^, so little is known about the effect of HT during anthesis on spikelet-opening of PTGMS rice lines. Being highly different from the conventional restore rice varieties, when the temperature is ≥23–24 °C, the male organ of PTGMS rice lines is totally sterile, so HT at anthesis mainly influence the spikelet-opening and later fertilization of female organ of PTGMS rice lines^3,13,20,36^ after pollinating with fertile pollen grains from restore line.

JA and MeJA are new plant hormones involved in the regulating plant growth and development and the response to environmental stresses^37–40^. They also play an important role in promoting flowering of cereals such as rice^38,41–47^. It has been reported that spikelet-opening in rice is mainly caused by lodicule expansion caused by water accumulation in the lodicules, and over 91% of the increase in lodicule fresh weight was due to water accumulation resulting from a sudden increase in osmotic pressure caused by starch and soluble sugar accumulation in lodicule cells^48–55^. The accumulation of osmotic regulation substances including potassium ion and other inorganic ion content along with soluble sugars in lodicules, had been reported mainly to be mediated by jasmonates in lodicules, eventually contributing to lodicule expansion and spikelet-opening^46,47^. The promotion of floret opening and the numbers of opening florets induced by external methyl jasmonate (MeJA) was correlated with the concentration applied in rice^56^.

The main purposes of this study were to investigate the effect of HT during anthesis on spikelet-opening of PTGMS rice lines and to understand the physiological mechanism underlying the effect. As the soluble sugars, starch, jasmonic acid (JA), methyl jasmonate (MeJA) and water content of the lodicules have been proposed to be closely associated with the spikelet-opening in rice, the above physiological traits and their relationships with the spikelet-opening and fertilization percentage were observed. Such a study would provide insight into the process in the spikelet-opening under HT during anthesis and explore the effective way to alleviate the damage of HT to PTGMS rice lines.

## Results

### Dynamics of spikelet-opening, length of spikelet-opening time and spikelet-opening angle

**Figure 1:**
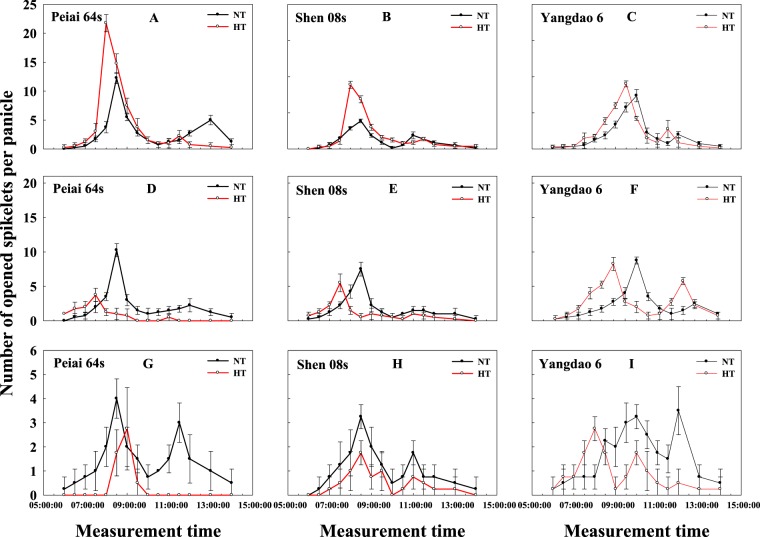
Effect of high temperature during anthesis on the dynamics of spikelet-opening of rice on the first day (**A**–**C**) and the second day (**D**–**F**) from the treatment and on the first day (**G**–**I**) after the treatment. The bars are SD of six biological replications. NT and HT represent normal temperature and high temperature, respectively.

Figure 1 illustrates the dynamics of spikelet-opening of three rice lines on the first day and the second day from the HT treatment and the first day after HT treatment. HT markedly enhanced spikelet-opening and increased the number of opened spikelets on the first day from the treatment, when compared with NT, and the increase in opened spikelets was more for the two PTGMS lines (Peiai 64S, 32.08%, Shen 08S, 38.74%) than for the restore line (Yangdao 6, 16.24%, Fig. 1A–C). However, HT decreased the number of opened spikelets of both PTGMS lines on the second day from the treatment, although the peak of spikelet-opening of the PTGMS lines under HT appeared earlier than under NT (Fig. 1D,E). For the restorer line, the peak of spikelet-opening also showed earlier under HT than under NT, but there was no significant difference in the number of opened spikelets between HT and NT (Fig. 1F). On the first day after treatment, HT substantially reduced the number of opened spikelets of the three test lines, with more reduction for the two PTGMS lines (Peiai 64S, 76.47%, Shen 08S, 53.97%) than for the restorer line (Yangdao 6, 43.75%, Fig. 1G–I).

**Figure 2:**
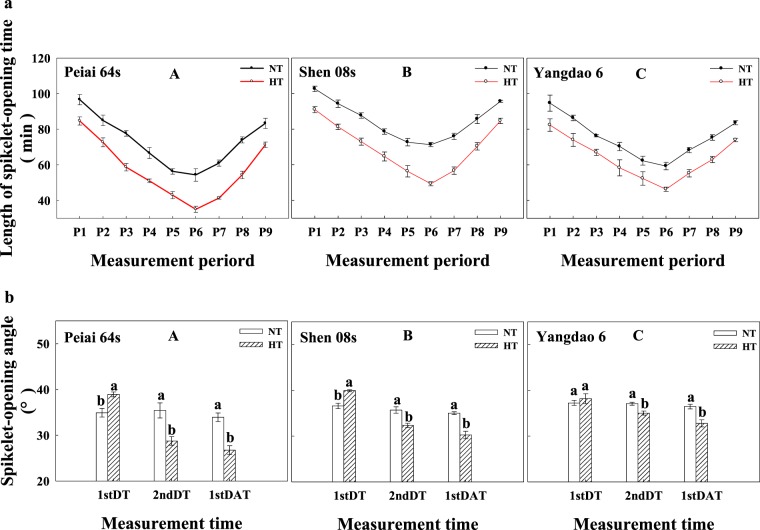
Effect of high temperature during anthesis on the length of spikelet-opening time (**a**) and spikelet-opening angle (**b**) of rice on the first day from the treatment. (**a**) Effect of high temperature during anthesis on the length of spikelet-opening time of rice on the first day from the treatment. (**b**) Effect of high temperature during anthesis on the spikelet-opening angle. The bars are SD of six biological replications. Different letters represent significant differences at *P* < 0.05 in the same line/variety. NT and HT represent normal temperature and high temperature, respectively. P1, P2, P3, P4, P5, P6, P7, P8 and P9 represent 7:00 h–8:00 h, 8:01 h–9:00 h, 9:01 h–10:00 h, 10:01 h–11:00 h, 11:01 h–12:00 h, 12:01 h–13:00 h, 13:01 h–14:00 h, 14:01 h–15:00 h, 15:01 h–16:00 h on the same day, respectively. 1stDT, 2ndDT and 1stDAT indicate the first day and the second day from the treatment and the first day after the treatment, respectively.

The length of spikelet-opening time for one spikelet was decreased under HT compared with that under NT, with more reduction for both PTGMS lines than for the restorer line, especially during 11:00–14:00 h (Fig. 2a,A–C). In contrast to the length of spikelet-opening time, the spikelet-opening angle of the two PTGMS lines was increased on the first day from the treatment, while it was decreased on the second form the treatment and on the first day after treatment, when the comparison was made between HT and NT (Fig. 2b,A–C). The increase and decrease in the spikelet-opening angle under HT was more for the two PTGMS lines than for the restorer line, indicating that PTGMS lines are more sensitive to HT during anthesis than the restorer line.

### Percentages of wrapped spikelets and opened spikelets

Percentage of wrapped spikelets and the percentage of opened spikelets of each rice line differed with temperature treatments (Table 1). Compared with NT, HT significantly increased the percentage of wrapped spikelets by 8.5% for Peiai 64S and by 3.9% for Shen 08S. However, HT showed no any effect on the percentage of wrapped spikelets for Yangdao 6 (Table 1), indicating again that the two PTGMS lines are more sensitive to HT than the restorer line. HT during anthesis significantly decreased the number and the percentage of opened spikelets, with more reduction for the two PTGMS lines (Peiai 64S, 18.62%, Shen 08S, 13.42% for the number of opened spikelets, Peiai 64S, 8.3%, Shen 08S, 5.0% for the percentage of opened spikelets) than for the restorer line (Yangdao 6, 5.56% and 4.9%, Table 1). The HT also significantly decreased spikelet number per panicle for both PTGMS lines, but HT did not affect the spikelet number for the restorer line (Table 1), suggesting that the two PTGMS lines are more sensitive to HT than the restorer line in terms of panicle-heading and spikelet-opening.

**Table 1:** Effect of high temperature during anthesis on the percentages of wrapped and opened spikelets in rice.

Line/variety	Treatment	% of wrapped spikelets	Spikelets per panicle	Opened spikelets per panicle	% of opened spikelets
Peiai 64S	NT	14.5 ± 0.48c	154 ± 1.5b	145 ± 2.0c	94.4 ± 1.0a
	HT	23.0 ± 0.83a	137 ± 1.5c	118 ± 1.5d	86.1 ± 0.2d
Shen 08S	NT	12.4 ± 0.38d	127 ± 2.5d	114 ± 1.5d	90.3 ± 1.5b
	HT	16.3 ± 0.47b	116 ± 2.5e	98.7 ± 3.0e	85.3 ± 2.5d
Yangdao 6	NT	0.0e	190 ± 2.6a	177 ± 2.5a	93.0 ± 0.6a
	HT	0.0e	190 ± 1.0a	167 ± 2.1b	88.1 ± 0.7c

NT and HT represent normal temperature and high temperature, respectively. Different letters within a column indicate significant difference at P < 0.05 (n = 6).

### Percentages of seed-setting and fertilization, seed yield, and their relationship with spikelet-opening characteristics

Similar to the percentage of opened spikelets, the percentages of seed-setting and fertilization, the seed yield of the two PTGMS lines hybridized with Yangdao 6, and the seed yield of Yangdao 6 were significantly decreased under HT, compared with those under NT (Table 2). The reductions were more for the two hybrids (Peiai 64S, 46.88%, Shen 08S, 48.94% for percentage of seed-setting; Peiai 64S, 45.91%, Shen 08S, 48.87% for the percentage of fertilization; Peiai 64S, 53.36%, Shen 08S, 51.07% for the yield) than for the inbred (the restorer line, 23.38%, 23.54%, 20.99%, for the three reductions). For example, the seed yield was reduced by 53.4% and 33.2% for the two hybrids of Peiai 64S × Yangdao 6 and Shen 08S × Yangdao 6, respectively, while the yield was reduced by 21.0% for Yangdao 6, relative to corresponding controls (NT). The reduction in the seed yield under HT was due mainly to the decreases in the seed-setting percentage and fertilization percentage. The 1000-grain weight showed no significant difference between HT and NT in the same hybrid or variety except Peiai 64S × Yangdao 6 which showed significant increase in 1000-grain weight under HT (Table 2).

**Table 2:** Effect of high temperature during anthesis on the seed yield and its components of rice.

Hybrid/variety	Treatment	Spikelets per pot	Seed-setting percentage (%)	Fertilization percentage (%)	10^3^-grain weight (g)	Seed yield (g pot^−1^)
Peiai 64S × Yangdao 6	NT	4462 ± 275.3a	25.6 ± 1.4d	28.1 ± 1.4e	19.4 ± 0.3c	22.3 ± 1.2d
	HT	3717 ± 260.0c	13.6 ± 0.3e	15.2 ± 1.2f	20.7 ± 0.1b	10.4 ± 1.0f
Shen 08S × Yangdao 6	NT	3075 ± 97.1d	51.9 ± 2.0c	53.0 ± 1.7c	17.6 ± 0.2c	28.0 ± 1.7c
	HT	2815 ± 11.2e	26.5 ± 1.0d	27.1 ± 1.5d	18.2 ± 0.1c	13.7 ± 0.5e
Yangdao 6	NT	4059 ± 95.8b	93.2 ± 2.1a	94.3 ± 0.9a	30.2 ± 0.1a	112.9 ± 4.0a
	HT	4128 ± 83.4b	71.5 ± 2.9b	72.1 ± 2.7b	30.6 ± 0.3a	89.2 ± 2.7b

NT and HT represent normal temperature and high temperature, respectively. Different letters within a column indicate significant difference at P < 0.05 (n = 5).

Correlation analysis showed that the length of spikelet-opening time, spikelet-opening angle, and the percentage of opened spikelets positively, whereas the percentage of wrapped spikelets negatively, correlated with the seed-setting percentage and the fertilization percentage (Table 3). The results imply that decreases in the length of spikelet-opening time, spikelet-opening angle and the percentage of opened spikelets and an increase in percentage of wrapped spikelets, especially for PTGMS lines, contribute to the reduction in the seed-setting percentage and the fertilization percentage, leading to the reduction in seed yields of the hybrids and the inbred under HT.

**Table 3:** Correlations of seed-setting and fertilization percentages with spikelet-opening characteristics.

Correlation with	Seed-setting percentage	Fertilization percentage
Percentage of opened spikelets	0.899^**^	0.916^**^
Percentage of wrapped spikelets	−0.964^**^	−0.974^**^
Length of spikelet-opening time	0.720	0.756^*^
Angle of spikelet-opening	0.748^*^	0.791^*^

**Figure 3:**
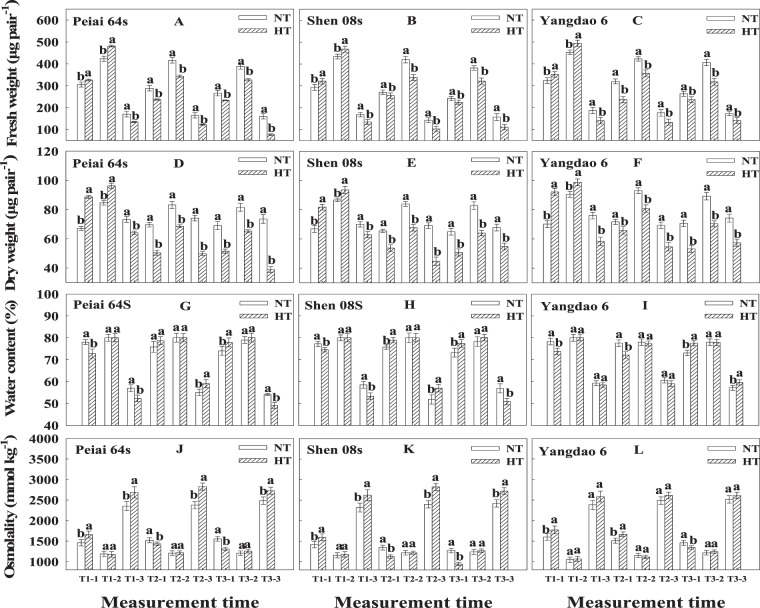
Effect of high temperature during anthesis on the lodicule fresh weight (**A**–**C**), dry weight (**D**–**F**), water content (**G**–**I**) and osmolality (**J**–**L**) of rice. The bars are SD of six biological replications. Different letters represent significant differences at *P* < 0.05 in the same line/variety. NT and HT represent normal temperature and high temperature, respectively. T1 and T2 are days on the first and second day of treatment, respectively, and T3 indicates the first day after treatment. The number of −1, −2 and −3 represents before and during spikelet-opening and after the close of spikelet, respectively.

Data for correlation analysis are from Tables 1and 2, Figs. 2 and 3. *and** represent significant at P < 0.05 and P < 0.01, respectively (n = 6).

### Lodicule fresh weight, dry weight, water content and osmolality

Under either NT or HT, lodicule fresh weight and dry weight were the highest during the spikelet-opening, followed by before spikelet-opening, and the lowest after the close of spikelet (Fig. 3A–F). Compared with NT, HT significantly decreased lodicule fresh weight and dry weight before and during spikelet-opening on the first and second day from the treatment and the first day after the treatment. Either fresh weight or dry weight after the close of spikelet was significantly lower under HT than under NT on all the measurement time (Fig. 3A–F).

Water content of lodicules varied with the time of spikelet-opening, the day of treatment, and rice lines (Fig. 3G–I). Generally, HT significantly decreased the water content before spikelet-opening on the first day from the treatment for all the three rice lines and after the close of spikelet on the first day from and after the treatment for the two PTGMS lines. There was no significant difference between NT and HT in the water content of lodicules during spikelet-opening on all the days and for all the lines. (Fig. 3G–I).

In a sharp contrast to the lodicule fresh weight and dried weight, the lodicule osmolality was the highest after the close of spikelet, followed by before spikelet-opening, and the lowest during spikelet-opening on all the days of treatment and for all rice lines (Fig. 3J–L). In comparison with NT, HT significantly increased the osmolality before spikelet-opening on the first day of treatment for all rice lines and after the close of spikelet for both PTGMS lines. No significant difference was observed during spikelet opening on all the days of treatment and for all the lines (Fig. 3J–L).

### Contents of soluble sugars, starch, ja, and meja in lodicules

**Figure 4:**
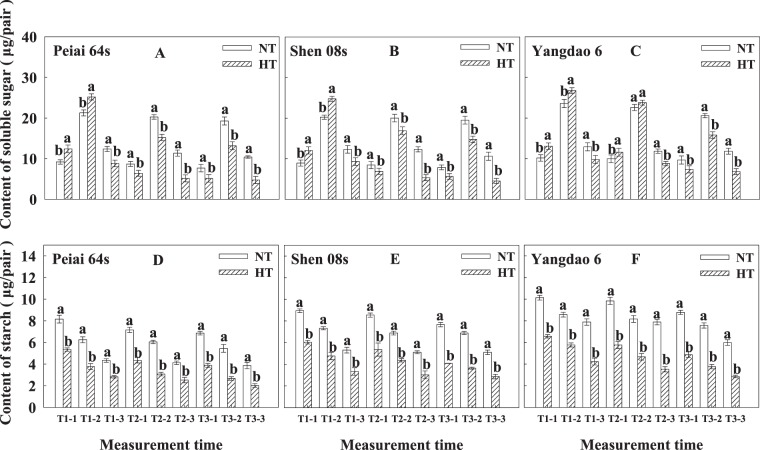
Effect of high temperature during anthesis on the contents of soluble sugars (**A**–**C**) and starch (**D**–**F**) in the lodicules of rice. The bars are SD of six biological replications. Different letters represent significant differences at *P* < 0.05 in the same line/variety. NT and HT represent normal temperature and high temperature, respectively. T1 and T2 are days on the first and second day of treatment, respectively, and T3 indicates the first day after treatment. The number of −1, −2 and −3 represents before and during spikelet-opening and after the close of spikelet, respectively.

The content of soluble sugars in lodicules was higher during spikelet-opening than before spikelet-opening or after the close of spikelet (Fig. 4A–C). HT significantly increased the content of soluble sugars before and during spikelet-opening and significantly decreased the content after the close of spikelet on the first day of treatment for all rice lines when compared with NT. The content of soluble sugars was lower under HT than under NT on the second day of the treatment and on the first day after the treatment for both PTGMS lines (Fig. 4A,B). For the restorer line and on the second day of treatment, the content of soluble sugars in lodicules was higher under HT than NT before spikelet-opening, and showed no significant difference between HT and NT treatments during spikelet-opening, and was lower under HT than under NT after the close of spikelet (Fig. 4C). HT significantly decreased the content of soluble sugars at the all times on the first day after the treatment for the restorer line (Fig. 4C).

In contrast to the increase in content of soluble sugars under HT before and during spikelet-opening on the first day of treatment, starch content in lodicules was significantly decreased under HT at all the measurement time, in comparison with that under NT (Fig. 4D–F). At the same measurement time, the starch content was higher for the restorer line than for the PTGMS lines.

**Figure 5:**
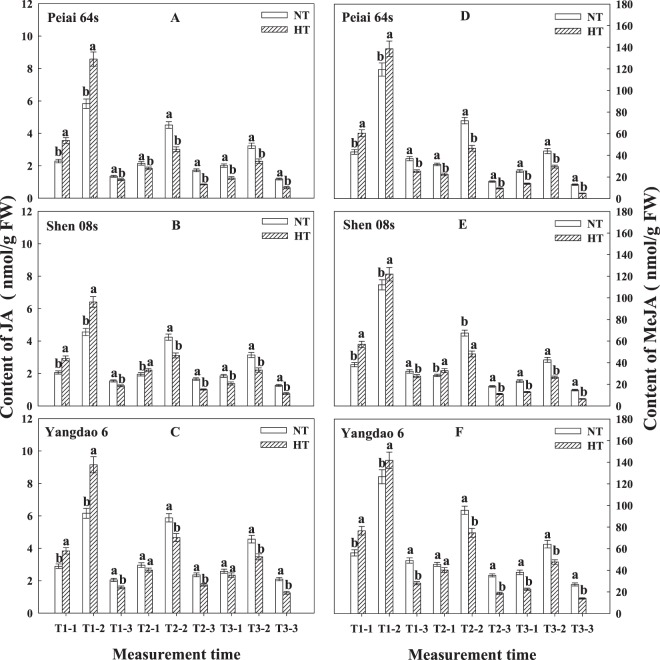
Effect of high temperature during anthesis on the contents of jasmonic acid (JA) (**A**–**C**) and methyl jasmonate (MeJA) (**D**–**F**) in the lodicules of rice. The bars are SD of three biological replications. Different letters represent significant differences at *P* < 0.05 in the same line/variety. NT and HT represent normal temperature and high temperature, respectively. T1 and T2 are days on the first and second day of treatment, respectively, and T3 indicates the first day after treatment. The number of −1, −2 and −3 represents before and during spikelet-opening and after the close of spikelet, respectively.

Similar to the content of soluble sugars in lodicules, JA content in lodicules was the highest during and before spikelet-opening on the first day of treatment, and it was increased during this period under HT compared with under NT (Fig. 5A–C). The JA content was low on the second day of the treatment and on the first day after treatment, and was decreased under HT. A similar changing pattern was observed for MeJA content in lodicules (Fig. 5D–F). However, MeJA content was much higher than JA content at the same measurement time. On the second day of the treatment and the first day after treatment, MeJA content was higher for the restorer line than for the PTMGS lines ((Fig. 5D–F).

### Correlation of lodicule traits with spikelet-opening

Before and during spikelet-opening, the lodicule traits including lodicule fresh weight, and the contents of soluble sugars and JA were significantly and positively correlated with the number of opened spikelets and the percentage of opened spikelets with *r* = 0.747* (*P* < 0.05) to 0.981** (*P* < 0.01) (Table 4). The lodicule osmolality and MeJA content before spikelet-opening were also significantly and positively correlated with the number of opened spikelets and the percentage of opened spikelets (*r* = 0.792* to 0.971**). Interestingly, lodicule dry weight both before and during spikelets-opening showed significant and positive correlation with the number of opened spikelets, but exhibited no significant correlation with the percentage of opened spikelets (*r* = −0.305 to −0.475, *P* > 0.05). In contrast, lodicule water content during spikelet-opening was significantly and negatively correlated with the number of opened spikelets (*r* = −0.854*, *P* < 0.05), but not significantly correlated with the percentage of opened spikelets before and during spikelet-opening and with the number of opened spikelets before spikelet-opening (*r* = −0.346 to −0.494, *P* > 0.05). The correlations of the number of opened spikelets and the percentage of opened spikelets with the starch content in lodicules before and during spikelet-opening and with lodicule osmolality and MeJA content during spikelet- opening were not significant (*r* = −0.660 to −0.194, *P* > 0.05) (Table 4). The results suggest that the reductions in lodicule fresh weight, contents of soluble sugars and JA before and during spikelet-opening and in the lodicule osmolality and MeJA content before spikelet-opening hinder the spikelet-opening under HT, especially for the PTMGS lines.

**Table 4:** Correlations of the number of and the percentage of opened spikelets with lodicule physiological traits.

Measurement time	Correlation with	Number of opened spikelets	Percentage of opened spikelets
Before spikelet-opening	Lodicule fresh weight	0.964**	0.787*
	Lodicule dry weight	0.860*	−0.470
	Lodicule water content	−0.494	−0.346
	Lodicule soluble sugar content	0.908*	0.912**
	Lodicule starch content	−0.497	−0.660
	Lodicule osmolality	0.854*	0.753*
	Lodicule JA content	0.965**	0.778*
	Lodicule MeJA content	0.971**	0. 792*
During spikelet-opening	Lodicule fresh weight	0.747*	0.941**
	Lodicule dry weight	0.924**	−0.305
	Lodicule water content	−0.854*	−0.475
	Lodicule soluble sugar content	0.981**	0.890**
	Lodicule starch content	−0.369	−0.538
	Lodicule osmolality	−0.523	−0.200
	Lodicule JA content	0.890*	0.748*
	Lodicule MeJA content	−0.501	−0.194

Data for correlation analysis are from Table 1 and Figs. 3, 4 and 5. *and** represent significant at P < 0.05 and P < 0.01, respectively (n = 6).

## Discussion

For a PTGMS rice line, the lemma open of a spikelet that protruded out the sheath of flag leaf, elongation of filaments, and exsertion of stigmas are necessary to fertilize with the pollens from a restorer rice line^55,57,58^. The results herein showed that four reasons might account for the decrease in seed yield when a PTGMS rice line was hybridized with a restorer rice line subjected to HT during anthesis. Firstly, the HT hindered the panicle exsertion from the sheath of flag leaf, which was evidenced with the increase in percentage of wrapped spikelets (Table 1). Secondly, the decrease in spikelet-opening angle under HT (Fig. 2b) resulted in a low probability for a PTGMS line to fertilize with the pollens from a restore line. Thirdly, HT significantly shortened the length of spikelet-opening time (Fig. 2a), which reduced the length of fertilization between a PTGMS line and a restorer line, and the last, but not the least, the failure in spikelet-opening under HT (Fig. 1) lost the possibility for a PTGMS line to receive the pollens from a restorer line for the fertilization. All which contributed to the reductions in seed-setting percentage, fertilization percentage, and seed yield when a PTGMS line hybridized with a restorer line (Table 2). Correlation analysis demonstrated that the seed-setting percentage and fertilization percentage were significantly and positively correlated with the percentage of opened spikelets, length of spikelet-opening time and spikelet-opening angle, but they were significantly and negatively correlated with the percentage of wrapped spikelets (Table 3). The results suggest that enhancing panicle exsertion form the sheath of flag leaf, extending the length of spikelet-opening time and increasing the spikelet-opening angle and the percentage of spikelet-opening under HT would be the important and effective approaches to improve the seed production for PTGMS rice lines when they are hybridized with restorer lines.

The decrease or increase in the number of opened spikelets (Fig. 1) was closely associated with the change in lodicule fresh weight, dry weight, water content before and during spikelet-opening (Fig. 3A–I,T1–1,T1–2,T2–1,T2–2,T3–1,T3–2) regulated by lodicule osmolality (Fig. 3J–L, T1–1,T2–1,T3–1), resulting from the accumulation of soluble sugars in lodicules (Fig. 4A–C, T1–1,T2–1,T3–1). There was also an interestingly secondary peak in each subfigure (Fig. 1A–F,H,I), which was consistent with previous reports^40,59^. However, only one peak was observed from Peiai 64S on the first day of HT treatment (Fig. 1G), which was probably due to the sharp and maximum decreases in soluble sugar and starch content in lodicules from Peiai 64S compared with other 2 testing lines (Fig. 4,T3), resulting in no more spikelets available for opening on another opening period.

As known from previous reports, the spikelet-opening of rice is mainly caused by the lodicule expansion at the base and inside of lemma and palea^46^, which was regulated by JA and MeJA in mediating osmotic substances including soluble sugars and starch contributing to lodicule expansion^47–50^. However, the present results showed that the lodicule osmolality was significantly increased (Figs. 3J–L,T1–3,T3–3), while the soluble sugar content was significantly decreased after spikelet-opening (Fig. 4A–C,T1–3,T3–3). The possible reason contributed to that “strange” change may resulted from decreased water content after spikelet-opening (Fig. 3G–I,T1–3,T3–3). Additionally, other osmotic substances including potassium ion and other inorganic ion content in lodicules^46,51–54^ may also compromise that change. Interestingly, on the second day of treatment, lodicule water content should be significantly increased after spikelet-opening under HT in comparison with that of under NT (Fig. 3G–I,T2–3), which could not be explained in terms of neither osmolality nor soluble sugar content. As far as I am concerned, maybe the speed gap between slower lodicule water withdraw speed, because of less energy opened spikelets, and faster soluble sugars consumption speed in spikelet-opening and respiration on the second day of HT treatment (Fig. 4A,B,T2-3), contributed to the this “strange” phenomenon.

It has been demonstrated that a fast increase in soluble sugar content usually accompany with a sharp decrease in starch content under heat stress^23,60^, just like the change in soluble sugar and starch content in lodicule at the temperature of 35 °C between 8:00- 9:00 am on the first day of HT treatment (Figs. 4A–F,T1–1,T1–2). The big increase in soluble sugar content in lodicule before and during spikelet-opening on the first day of HT treatment lead to a bigger spikelet-opening angle. However, with the extension of HT treatment time, the less soluble sugar content in lodicules (Fig. 4A–C,T1–3,T2-T3) due to the much consumption in spikelet-opening and respiration^61,62^, resulted in smaller spikelet-opening angle (Fig. 2, b2ndDT, 1stDAT) caused by a big shortage in osmotic substances for proper osmolality inducing lodicule expansion (Figs. 3J–L,T2–1 and T3–1). What’s more, the sharp decrease in soluble sugar content after spikelet-opening (Figs. 4A–C,T1–3,T2–3 and T3–3) notably accelerated the process of lodicule withering, eventually resulting in shortened the length of spikelet-opening time (Fig. 2a).

Further, the increases or decreases in opened spikelets of PTMGS lines were more than that of restore line under HT (Fig. 1), which was in accordance with the changes in lodicule fresh weight, dry weight, water content (Fig. 3A–I), which was regulated by lodicule osmolality before spikelet-opening (Fig. 3J–L). On the other hand, the increases or decreases in lodicules soluble sugar, starch, JA and MeJA content (Figs. 4, 5) contributed the change in osmolality, ultimately resulting in the increases or decreases in opened spikelets. According to previous report, the male sterile line Zhenshan 97A flowered later and closed later in comparison with male fertile line Zhenshan 97B, caused by fewer vascular bundles in male sterile line^46^, which was different from our study indicating that PTMGS lines flowered earlier and closed faster than restore line. While this spikelet-opening phenomenon was consistent with later study showing that glume closure was negatively correlated with soluble sugars in lodicules and male sterile line Guangzhan 63S flowering earlier than male fertile line 9311 in 2015, due to the more abundant vascular bundles in male sterile line Guangzhan 63S^23^. It has been proved that abundant vascular bundles were favored for the rapid absorption and release of water in lodicules during the opening and closing of the lemma and palea^23,53^. Our study showed that Peiai 64S and Shen 08S had more abundant vascular bundles than Yangdao 6 (Data not shown in this manuscript), indicating that the abundances in vascular bundles along with the characteristics in lodicule physiological traits such as soluble sugars was the main reason contributing to the sensitivity of PTMGS lines in spikelet-opening.

The results showed that the changes in contents of JA and MeJA in the lodicules of rice, especially for the PTMGS lines, were closely associated with those in spikelet-opening under HT, that is, the higher the contents of JA and MeJA, the more the number of opened spikelets (Figs. 1 and 5). The JA content before and during spikelet-opening, and MeJA content before spikelet-opening significantly and positively correlated with the number and the percentage of open spikelets (Table 4). The results imply that both JA and meJA in lodicules, especially JA, respond to HT during anthesis and play a vital role in regulating spikelet-opening of rice. The mechanism in which JA and MeJA regulate spikelet-opening, especially for PTGMS lines, was not investigated in this study. There are reports showing that, in the lodicules of male sterile rice lines, endogenous JA could mediate the accumulation of osmotic regulation substances in the lodicules, and consequently, regulate lodicule expansion and spikelet-opening^41,44,45,47,56^. It is observed that application of MeJA to the plants of male sterile rice lines subjected to HT during anthesis could significantly reduce the contents of H_2_O_2_ and O^2^, and increase the activities of catalase and peroxidase and the content of soluble sugars in the lodicules, and thereby increase the percentage of opened spikelets^63^. Therefore, we argue that JA and MeJA in lodicules respond to HT during anthesis and enhance spikelet-opening through increases in the antioxidant ability and osmotic substances there. Further investigation is needed to better understand the mechanism in which JA and MeJA regulate spikelet-opening in the lodicules of rice, especially for PTGMS rice lines subjected to HT during anthesis. Jasmonic acid (JA) and its methyl ester can also induce ethylene production^64^, and ethylene is crucial for reactive oxygen species (ROS) generation in plants^65–68^. Both ethylene and ROS can have positive effects on plant growth and development at a probably low concentration^68^, although they are often considered to be a plant stress hormone and highly reactive molecules, capable of causing oxidative damage to protein, DNA and lipids when increasing to excessive levels under various biotic and abiotic environmental stresses^69–72^. Although little is known about the cross-talk between ethylene and antioxidant enzymes in mediating ROS generation under stress environment. So maybe the application of ethylene at proper concentration could also mediated the damage caused by ROS under HT and promote the spikelet-opening, it worth further study.

It is noteworthy that an increase in the percentage of wrapped spikelets under HT was another important reason for the decreases in the number and percentage of opened spikelets for PTGMS lines, and no any effect of HT on the spikelet exsertion from the sheath of flag leaf was observed for the restorer line (Table 1). The mechanism in which HT hinders panicle or spikelet exsertion from the sheath of flag leaf in PTGMS lines and the way to eliminate such an obstacle merits further research.

## Conclusion

HT during anthesis significantly increased the percentage of wrapped spikelets and decreased the spikelet-opening angle, the length of spikelet-opening time, and the number and the percentage of opened spikelets of rice, especially for PTGMS lines, leading to reductions in the percentages of seed-setting and fertilization and seed yield of the PTGMS lines that were hybridized with the restorer line. Decreases in the fresh weight and the contents of soluble sugars, JA and MeJA in lodicules before and during spikelet-opening accounted for decreases in the length of spikelet-opening time, the length of spikelet-opening time, and the number and the percentage of opened spikelets. Further research is needed to better understand the mechanism in which HT during anthesis affects the spikelet-opening and the panicle exsertion from the sheath of flag leaf and to explore the way to eliminate the adverse effect on the PTGMS rice lines.

## Materials and Methods

### Plant materials and cultivation

Experiments were conducted at the research farm of Yangzhou University, Jiangsu Province, China (32°30′N, 119°25′E, 21 m altitude) during the rice growing season (May–October) in 2018. Two PTGMS rice (*Oryza sativa* L.) lines, Peiai 64S, Shen 08S, and one restorer line Yangdao 6 (as a normal pollen donor and a conventional rice variety), were used. The sowing date of the PTGMS lines was on 15 May, and that of the restorer line was on 5, 10, 15 and 20 May, respectively, so that the flowering period of the restorer line could meet that of the PTGMS lines. The seedlings were grown in the paddy field, and then thirty-day-old seedlings were transplanted to porcelain pots with three hills per pot and two seedlings per hill. Each porcelain pot (25 cm in diameter, 30 cm in height and 14.72 L in volume) was filled with 15 kg sandy loam soil [Typic fluvaquents, Etisols (U.S. taxonomy)] with organic matter 24.5 g kg^−1^, alkali-hydrolysable N 108 mg kg^−1^, Olsen-P 34.7 mg kg^−1^, exchangeable K 68.1 mg kg^−1^. A 2–3 cm water layer above the soil surface was kept in pots and the soil pH was adjusted to 6.0 with 0.1 mol L^−1^ HCl for 3 weeks before rice seedlings were transplanted into pots. At 3 days before transplanting, 1 g N as urea [(NH_2_)_2_CO] and 0.5 g K as KCl were mixed into the soil in each pot. N as urea was also applied at mid-tillering (0.5 g pot^−1^) and at panicle initiation (1 g pot^−1^) stages. Two PTGMS rice lines headed on 1 and 5 August, respectively, for Peiai 64S and Shen 08S, and were hybridized with the restorer line during anthesis. The hybrid seeds were harvested on 29 and 30 October.

### High temperature treatment

When approximate 50% of the plants were heading from the sheaths of flag leaves, the plants heading on the same day were chosen and tagged and then were moved into four phytotrons (AGC-MR, Zhejiang Qiushi Environment Co., Ltd, Zhejiang, China) for temperature treatments. Each phytotron was 6.0 m in length, 3.2 m in width and 2.4 m in height, and was equipped with intelligent controlling system of temperature, light density, CO_2_ concentration, and humidity. Both normal temperature (NT, the control) and high temperature (HT) were set in accordance with the diurnal variation of air temperature at local weather conditions. During the period of 06:01–08:00, 08:01–10:00, 10:01–14:00, 14:01–16:00, 16:01–18:00, 18:01–20:00 and 21:00–06:00 h, the temperature (°C) was 26, 29, 32, 30, 28, 26, and 24, respectively, for the NT, and was 30, 35, 39, 35, 32, 30, and 26, respectively, for the HT treatment. The light density for both NT and HT was 800, 1000, 1000, 1000, 800, and 100 µmol m^−2^ s^−1^, respectively, for the first 5 period mentioned above. The relative humidity and CO_2_ concentration were kept at 75% and 380 ± 20 µmol mol^−1^ for both NT and HT during the treatment, respectively. The period of temperature treatment lasted for 48 h from 06:00 h on the first day to the 06:00 h on the third day of the treatment. During anthesis, the pollens from the restorer line under NT were fully fertilized with pistils of the PTGMS lines under both NT and HT with the help of manual pollination. Each treatment for each rice line had 40 pots as replications.

### Sampling and determination

Three hundred tagged panicles were sampled from each treatment at each day during the temperature treatment. And then they were used for the measurements of spikelet-opening characteristics including the number and the percentage of opened spikelets, percentage of wrapped spikelets (wrapped by the sheath of flag leaf), length of spikelet-opening time, and the physiological traits of lodicules including fresh and dry weight, water content, osmolality, and contents of soluble sugars, starch, jasnonic acid (JA) and methyl jasmonate (MeJA).

### Percentages of opened and wrapped spikelets

The number of opened spikelets on the panicle headed out from the sheath of flag leaf with an angle ≥10° between the palea and lemma was marked and counted at every 30 min from 06:00 to 15:00 until the spikelets were fully opened. The total number of opened spikelets during the 2-day treatment period and on the first day after treatment, and the number of wrapped spikelets during the heading period were counted. Each treatment had six replications. The percentage of opened spikelets and the percentage of wrapped spikelets on a panicle were calculated using following formulas:

Percentage of opened spikelets (%) = the number of opened spikelets with an angle ≥10° on a panicle headed out from the sheath of flag leaf/the total number of spikelets (opened spikelets + closed spikelets) on a panicle that headed out form the sheath of flag leaf × 100

Percentage of wrapped spikelets (%) = the number of spikelets on a panicle wrapped by the sheath of flag leaf/the total number of spikelets on a panicle × 100

### Length of spikelet-opening time and spikelet-opening angle

Every six opening spikelets from each treatment were chosen and marked to investigate the length of spikelet-opening time and spikelets-opening angle, respectively. It’s only 2–3 min from the beginning of lemma and palea opening, just a few seconds after visible expansion at the base of spikelet and light yellow visible color through the middle of spikelet due to the mature pollen grains, to the largest spiklet-opeing angle, when filaments stretch out of the spikelet. The process of spikelet-opening was photographed by mobile phone at every 30S until the filament totally stretched out of a spikelet, then the interval of photographing changed into 5 min until the close between the palea and lemma. The length of spikelet-opening time was calculated according to the pictures taken during the spikelet-opening process. The spikelet-opening angle was carefully measured using the image editing software (ZEN 2012, Carl Zeiss Microscopy Gmbh, ZEISS, Germany). The biggest angle between palea and lemma was recorded as the spikelet-opening angle.

### Lodicule fresh and dry weight, water content and osmolality

Six hundred spikelets from each line and each treatment were successively sampled before and during spikelet-opening and after the close of spikelets during the 2-day treatment period and on the first day after treatment. Sampled spikelets were put in the box filled with ice, and ten pairs of fresh lodicules in 10 spikelet was quickly took out using tweezers and weighed. About 4–5 mg lodicules (ten pairs) were dried in an oven for the measurement of dry weight. Eight pairs of lodicules from each treatment were used for determining osmolality on a pressure osmometer (Vapro5520, Wescor, Logan, UT, USA). Each treatment had six biological replications. The rest of sampled lodicules were stored in a refrigerator at −80 °C pending for the measurement of soluble sugars, starch, JA and MeJA. The water content of lodicules was calculated with the formula:

$${\rm{\text{Water content of lodicules}}}( \% )=(\text{lodicule fresh weight}-\text{lodicule dry weight})/\text{lodicule fresh weight}\times 100$$

### Contents of soluble sugars, starch, ja and meja in lodicules

The contents of soluble sugars and starch in lodicules were determined as described by Yoshida *et al*.^73^ and Pucher *et al*.^74^, respectively. Fifty pairs of lodicules with three biological replications from 150 spikelets of each line from each treatment were isolated and dried in an oven for the measurement of soluble sugar and starch content. Each sample consisted of 50 pairs of lodicules were extracted with 5 mL 80% (v/v) ethanol by incubating for 30 min at 80°C Cool to room temperature and then centrifuging at 2000 rpm for 20 min, and the extraction step was repeated twice. All the supernatant was combined and 2 mL of supernatant above and 4 mL 0.2% enthrone reagent were mixed and boiled for 15 min. Subsequently quantified using the anthrone colorimetric method at 620 nm when the mixture were cooled to room temperature. The lodicule residue was dried at 60◦C and then boiled in 2 mL distilled water for 20 min, then digested in 2 mL 9.2 mol·L^−1^ HClO_4_ for 10 min with continuous shaking. After centrifuging at 2000 rpm for 20 min, apply 4.6 mol·L^−1^ HClO_4_ in lodicule residue for another starch extraction. The starch in the supernatant was determined using the anthrone colorimetric method.

The extraction of JA and MeJA in the lodicules was modified from Liu *et al*.^75^. Briefly, approximately 50–70 mg of fresh lodicules with 3 biological replications of each treatment was frozen in liquid N_2_ and was ground by a tissue crusher (MM400, Retsch Corp, Haan, Germany), then was added with 400–500 μL of methanol, and they were mixed and kept at 4 °C overnight, then centrifuged at 4800 × *g* for 10 min. The supernatant was transferred to a 2 mL glass tube and the residue was re-extracted with 200 μL of methanol. Before the supernatant was applied onto the Waters Sep-pak C18 cartridge, the cartridge was washed with 200 μL of 20% methanol and 250 μL of 30% methanol, respectively. Finally, the cartridge was eluted with 300 μL of 100% methanol. The eluted solution was collected to pass through an organic microporous membrane of 0.22 μm. Then JA and MeJA were quantified using a liquid chromatography tandem mass spectrometry (LC-MS-MS) system (Thermo Fisher Scientific, Waltham, MA, USA), according to the method of Liu *et al*.^33^. The optimized condition for the analysis of JA and MeJA was ion pray voltage 4800 V, atomization gas 30 mL min^−1^, auxiliary gas 2 mL min^−1^, transfer capillary temperature 380 °C, and offset lens voltage 77 V; the main fragment ion (m/z): JA (59.1/165.1), MeJA (151); collision energy 15 e V; signal acquisition 15–19 min.

### Final harvest

Plants from five pots in each treatment were harvested at maturity for the determination of the number of spikelets per pot, the seed-setting percentage, fertilization percentage, grain weight, and seed yield. The seed-setting percentage was defined as the filled grains (specific gravity ≥1.06 g cm^−3^) as a percentage of total number of spikelets, and the fertilization percentage was defined as the fertilized spikelets as a percentage of the total number of spikelets.

### Statistical analyses

The results were analyzed for variance using the SAS/STAT statistical analysis package (version 9.12, SAS Institute, Cary, NC, USA). Data from each sampling time were analyzed separately. Means were tested by least significant difference at the *P* = 0.05 level (LSD_0.05_). Correlation analysis was used to evaluate the relationships between the spikelet-opening characteristics and the physiological traits of lodicules.

## Data Availability

The authors declare that the datasets generated during and analysed during the current study are available from the corresponding author on reasonable request.
